# Antimicrobial Activity of Green Synthesized Silver and Copper Oxide Nanoparticles against the Foodborne Pathogen *Campylobacter jejuni*

**DOI:** 10.3390/antibiotics13070650

**Published:** 2024-07-14

**Authors:** Daniel Rivera-Mendoza, Beatriz Quiñones, Alejandro Huerta-Saquero, Ernestina Castro-Longoria

**Affiliations:** 1 Department of Microbiology, Center for Scientific Research and Higher Education of Ensenada (CICESE), Ensenada 22860, Mexico; riveramd@cicese.edu.mx; 2 Produce Safety and Microbiology Research Unit, Western Regional Research Center, Agricultural Research Service, U.S. Department of Agriculture (USDA), Albany, CA 94710, USA; beatriz.quinones@usda.gov; 3 Department of Bionanotechnology, Center for Nanoscience and Nanotechnology, Universidad Nacional Autónoma de México, Ensenada 22860, Mexico

**Keywords:** antibacterial activity, *Campylobacter*, food safety, foodborne pathogen, silver, copper oxide, nanoparticles, green synthesis

## Abstract

*Campylobacter jejuni* is a major cause of global foodborne illnesses. To develop alternative antimicrobial strategies against *C. jejuni*, this study designed and optimized the green synthesis of metallic nanoparticles (NPs) with intracellular components of the medicinal fungus *Ganoderma sessile* to provide the needed reducing and stabilizing agents. NPs were characterized by transmission electron microscopy and dynamic light scattering, and the quasi-spherical NPs had sizes of 2.9 ± 0.9 nm for the copper oxide NPs and 14.7 ± 0.6 nm for the silver NPs. Surface charge assessment revealed zeta potentials of −21.0 ± 6.5 mV and −24.4 ± 7.9 mV for the copper oxide and silver NPs, respectively. The growth inhibition of *C. jejuni* by the NPs occurred through attachment to the outer cell membrane and subsequent intracellular internalization and resulted in minimum inhibitory concentrations of the silver NPs at 6 µg/mL and copper oxide NPs at 10 µg/mL. On the other hand, a differential ROS production caused by silver and copper NPs was observed. In summary, this research presents the first demonstration of using green synthesis with the medicinal fungus *G. sessile* to produce metallic NPs that effectively inhibit *C. jejuni* growth, providing a sustainable and effective approach to the traditional use of antimicrobials.

## 1. Introduction

Nanotechnology stands at the forefront of scientific advancements, providing many applications across diverse sectors like medicine, agriculture, and environmental remediation. The synthesis of nanoparticles (NPs), with their unique physicochemical properties, has garnered considerable attention for its potential benefits, particularly in human health [1,2,3,4]. These applications span from drug delivery systems to the development of chemically functionalized dendrimers, acting as molecular building blocks for gene therapy agents and exerting antimicrobial effects against different bacterial pathogens that are multidrug-resistant [3,5,6]. For these applications, green chemistry has emerged as a focal point in NPs synthesis, drawing interest for their eco-friendly and sustainable characteristics [7]. In particular, the use of ‘bio-factories’ such as fungi and plant extracts for providing the reducing and stabilizing agents needed for a successful NP synthesis represents a novel and alternative approach to the use of green chemistry methodologies [7,8]. When compared to conventional methods [9], green synthesis techniques thus offer significant advantages, since these methods are more cost-effective and environmentally benign and require milder reaction conditions for the synthesis of NPs with enhanced biocompatibility, stability, and acceptable uniformity [1].

Among the fungi used in nanotechnology, *Ganoderma* is a large and diverse genus of wood decay fungi and has been used as traditional medicine for thousands of years in many Asian countries. In countries like China, *Ganoderma* has been employed to treat diseases such as hepatitis, chronic bronchitis, gastritis, tumor growth, and immunological disorders, among others [10]. More recently, the pharmaceutical industry has shown great interest in these fungal species due to their bioactive compounds and therapeutic properties, including immunomodulatory effects, anti-tumor activities, cardiovascular health promotion, and anti-inflammatory properties [11,12]. Within the *Ganoderma* species, *Ganoderma lucidum* is the best characterized and occurs endemically in Europe and China. In recent years, the use of *G. lucidum* has been integrated with the development of nanotechnology for advancing biomedical applications, since the health benefits of *Ganoderma* have provided added value in the context of NP synthesis by utilizing its compounds with bioactive properties [10,12,13,14,15,16,17,18]. Characterization studies of this globally distributed fungi have reported *Ganoderma sessile*, a species found in Mexico and the United States [11,19]. In particular, *G. sessile* has been shown to be a high-yield producer of polysaccharides [20], and these biopolymers are one of the requirements used as protective colloids which help in the synthesis of the active adsorbate layers of the NPs by stabilizing the particles sterically and electrostatically [21]. Moreover, previous reports have documented the synthesis of metallic NPs using *G. sessile*, and these findings have provided compelling evidence on the potential use of these fungi-derived particles against enteric and opportunistic bacterial pathogens [22,23].

Given that foodborne illnesses loom globally, the use of NPs has gained significant attention for their potential applications in food safety [22,23,24,25,26]. Among foodborne pathogens, *Campylobacter jejuni* is considered a major cause of global foodborne illnesses [27,28,29]. Ingestion of contaminated food, notably undercooked poultry or unpasteurized milk, is a significant source of *Campylobacter* infection in humans, leading to symptoms such as diarrhea, abdominal pain, fever, and vomiting. Some rare cases of *C. jejuni* infection may result in sequels like Guillain–Barré syndrome, a neurological disorder [28,30]. Integrating nanotechnology in combating this foodborne pathogen holds promise for enhancing food safety and reducing the incidence of *Campylobacter*-related infections. Given that the impact of *Campylobacter*-related infections on human health and the economy is substantial, effective control and mitigation strategies are thus necessary to prevent the transmission of *Campylobacter* in the food chain. To address these challenges for food safety, the present study aimed to apply and adapt the use of green synthesis of silver and copper oxide NPs by using the intracellular components of *G. sessile* to inhibit the growth of *C. jejuni*.

## 2. Results

### 2.1. Optimized Biosynthesis of Silver and Copper Oxide NPs Derived from G. sessile

**Figure 1:**
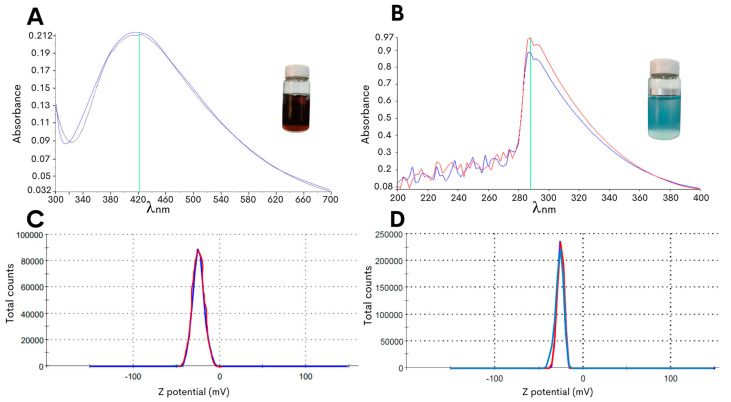
Characterization of the biosynthesized NPs using *G. sessile* intracellular extract. The lines of different colors in the graphs are the repetitions made when taking the measurement. The absorbance values in the ultraviolet-visible spectra were measured for the silver NPs (**A**) or the copper oxide NPs (**B**). An assessment of the particle surface charge was based on the zeta potential measurement of the silver NPs (**C**) or the copper oxide NPs (**D**).

In the present study, an optimized methodology was developed for the synthesis of copper oxide or silver NPs with the edible fungus *G. sessile* by controlling the changes in pH to obtain stable NPs with an improved efficacy against *C. jejuni*. As the starting material for the green synthesis of the NPs, the fungal mycelia was macerated to obtain an aqueous intracellular extract, and the extract was then mixed with the metal precursor salts (see Section 4), resulting in a solution pH of approximately 3 to 4. As shown in Figure 1A, the synthesis method of the silver NPs resulted in a reaction color change to dark brown after 72 h of incubation with a peak absorbance at 420 nm. Moreover, the adjustment to pH 8.0 resulted in a change in the color of the reaction mixture to light blue for the copper oxide NPs, and this reaction color intensified after 72 h of incubation at 60 °C with the precursor salt and had the highest absorbance peak at 290 nm (Figure 1B).

**Figure 2:**
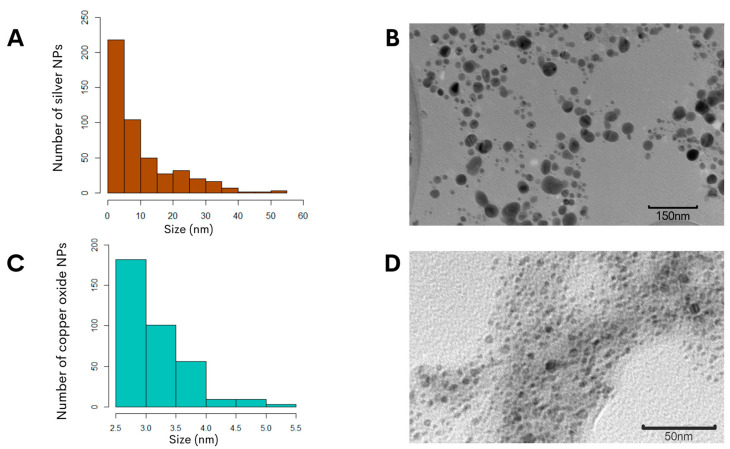
Morphological assessment of the biosynthesized NPs using *G. sessile* intracellular extract. The size distribution histogram of silver NPs (**A**) or copper NPs (**C**) indicated the range of the particle sizes. Representative transmission electron microscopy images of silver NPs (**B**) or copper NPs (**D**) revealed the morphology and sizes of the NPs.

To further characterize the biosynthesized NPs, the zeta potential measurement was determined in the aqueous solution to assess the surface charge of the NPs. The analysis revealed that the NPs had zeta potentials of −21.0 ± 6.5 mV for the copper oxide NPs and −24.4 ± 7.9 mV for the silver NPs (Figure 1C,D), and this significant negative charge indicated that the synthetized particles were electrically stabilized and would not result in aggregation or agglomeration [31,32]. Furthermore, analysis of representative transmission electron microscopy images demonstrated that the silver NPs were of small sizes, and quantification of the particle size distribution showed that the average diameter was 14.7 ± 0.6 nm (Figure 2A). Subsequent characterization of the copper oxide NPs revealed that their sizes were smaller than 10 nm, with an average size diameter of 2.9 ± 0.9 nm (Figure 2C).

### 2.2. Antimicrobial Effects of the Silver and Copper Oxide NPs against the Foodborne Pathogen C. jejuni

**Figure 3:**
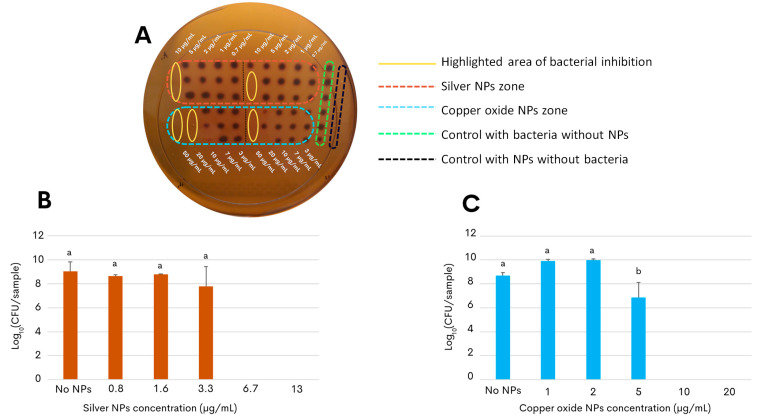
Inhibition effect of green synthesized NPs against *C. jejuni* strain ATCC 33560. (**A**) Initial approach to determine the approximate inhibitory concentrations of NPs against *C. jejuni*; area of bacterial inhibition is highlighted in yellow ovals with concentrations of 10 μg/mL for silver NPs (orange rectangle) and 20 μg/mL for copper oxide NPs (blue rectangle). (**B**,**C**) Colony-forming units of *C. jejuni* on selective solid media determined after 48 h of incubation under micro-aerophilic conditions in the presence of either silver NPs (**B**) or copper oxide NPs (**C**). Cultures of *C. jejuni* in the absence of NPs correspond to the control condition, shown as No NPs. The results are the mean and standard deviations of triplicate experiments. Bars with different lowercase letters represent statistically significant differences, as computed by one-way ANOVA and Tukey’s post hoc test (*p*-value < 0.05).

As a proof of concept for developing an antimicrobial application, the present study further assessed the nanotoxicity of the *G. sessile*-derived metallic NPs on the growth and survival of the foodborne pathogen *C. jejuni* strain ATCC 33560. The result of the first approach to determine the antibacterial activity of both types of NPs showed that low concentrations of the NPs were sufficient to inhibit the bacterial growth of *C. jejuni*, as seen in Figure 3A. Within the yellow ovals, it can be observed that the bacteria did not grow at concentrations of 10 μg/mL for silver NPs and concentrations of 20 μg/mL for copper oxide NPs. The following experimental approach aimed to determine the effective concentration of the silver and copper oxide NPs that would inhibit the growth of *C. jejuni* in liquid broth after incubation for 48 h (minimal inhibitory concentration, MIC). Quantification of the colony-forming units on selective solid media revealed that the growth-inhibitory effect on *C. jejuni* by the copper oxide NPs had a slightly more extended concentration range when compared to the silver NPs (Figure 3). In particular, the MIC of silver NPs was found at 6.7 μg/mL (Figure 3B) and that of the copper oxide NPs at a concentration above 10 μg/mL (Figure 3C), demonstrating their efficacy at a relatively low concentration.

**Figure 4:**
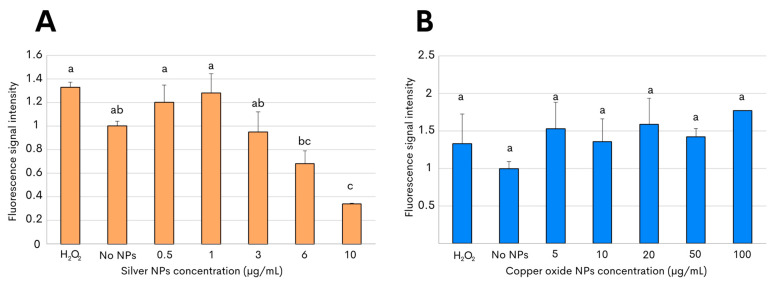
ROS production in *C. jejuni* strain ATCC 33560 after exposure to metallic NPs. Fluorescence levels were measured after incubation with 2′,7′-dichlorofluorescein diacetate to evaluate ROS generation in *C. jejuni* by either silver NPs (**A**) or copper oxide NPs (**B**). Incubation with 1 mM H_2_O_2_ or no NPs corresponded to the positive and negative controls, respectively. The results shown are the mean and standard deviations of triplicate experiments. Bars with different lowercase letters represent statistically significant differences, as determined by performing a one-way ANOVA with Tukey’s post hoc test (*p*-value < 0.05).

To subsequently characterize the nanotoxicity of the biosynthesized NPs, the production of reactive oxygen species (ROS) was then evaluated in the present study. Oxidative stress is considered to be an aftereffect of the interaction between metallic NPs and bacterial cells, and the release of the free radicals can thus have an antimicrobial effect on the targeted bacterial pathogen due to the injury caused by the NPs [33,34]. The assessment of oxidative stress in *C. jejuni* after exposure to both metallic NPs was determined by measuring the fluorescence emitted by 2′,7′-dichlorofluorescein diacetate, which oxidizes in the presence of reactive species [34,35]. A significantly increased level of ROS generation in *C. jejuni* was observed after incubation with 1 µg/mL with the silver NPs. However, reduced levels of ROS, significantly different from those detected in *C. jejuni* control cells, were the result after incubation, with even higher concentrations of silver NPs above 3 µg/mL (Figure 4A). By contrast, the assessment of oxidant formation in *C. jejuni* after incubation with copper oxide NPs exhibited ROS generation at levels that were similar to those measured after incubation in the absence of NPs (Figure 4B). In addition, no significant differences in the ROS generation were observed after the 3 h of incubation with 2′,7′-dichlorofluorescein diacetate in the present of the copper oxide NPs at any of the tested concentrations, ranging from 5 to 100 µg/mL (Figure 4B). For all experiments, 1 mM H_2_O_2_ was used as the positive control.

A bacterial viability test was conducted where *C. jejuni* was incubated for 24 h with the minimum inhibitory concentrations of both types of NPs, specifically 6.7 µg/mL of silver NPs and 10 µg/mL of copper oxide NPs. Samples were taken at 1 h, 3 h, 4 h, 6 h, and 24 h to assess bacterial growth. The results indicated that, with silver NPs, bacterial growth ceased after 3 h. In contrast, with copper oxide NPs, bacterial growth was observed up to 6 h, but no bacterial growth was detected after 24 h. This suggests that, while silver NPs exhibit a more immediate bactericidal effect, copper oxide NPs have delayed, but ultimately effective, antibacterial action.

### 2.3. Visualization of the Localized Interactions between the G. sessile-Derived Metallic NPs and C. jejuni

**Figure 5:**
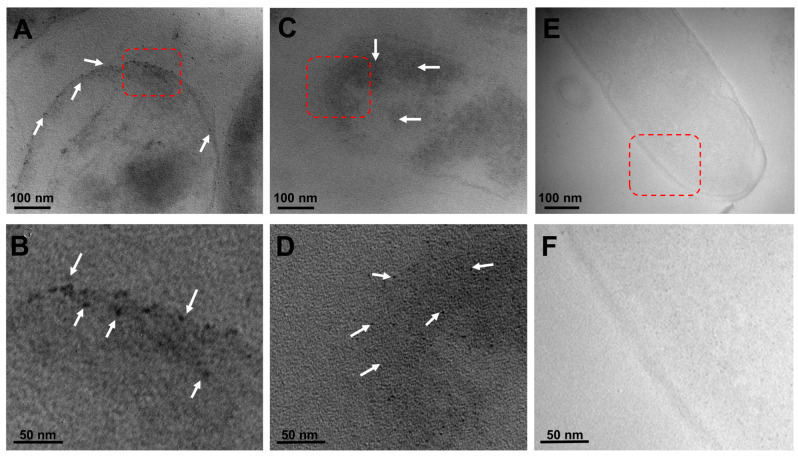
Localized interactions of *G. sessile*-derived metallic NPs with *C. jejuni*. Representative transmission electron microscopy images of *C. jejuni* strain ATCC 33560 demonstrate the specific localization of the silver NPs (**A**) with a red-outlined magnified section (**B**) or the localization of the copper oxide NPs (**C**) with a red-outlined magnified section (**D**). The white arrows indicate the metallic NPs. As negative controls, representative images of *C. jejuni* without metallic NPs incubation (**E**) with a red-outlined magnified section (**F**).

To further characterize the inhibitory effect of the green-synthetized metallic NPs against *C. jejuni*, transmission electron microscopy was then employed in conjunction with the typical sample preparation steps, involving fixation, resin infiltration, and generation of 50–70 nm sections [36,37], to better visualize the localized interactions between the metallic NPs and this foodborne bacterial pathogen. As shown in Figure 5, representative microscopy images illustrated the interaction of both types of metallic NPs, corresponding to black dots on the microscopy image, in *C. jejuni*. In particular, the images demonstrated the presence of silver NPs interacting with the outer cell membrane of *C. jejuni* (Figure 5A, white arrows), and the outer membrane attachment of the silver NPs was clear in a higher-magnification image (Figure 5B, white arrows). Similarly, examination of the interaction between the copper oxide NPs and *C. jejuni* revealed a membrane association of the NPs (Figure 5C). Subsequent microscopy analysis of the dissections also demonstrated an apparent internalization of the copper oxide NPs (Figure 5D, white arrows). As negative control conditions, Figure 5E,F correspond to representative images of *C. jejuni* without *G. sessile*-derived metallic NPs, indicating that the observed metallic NPs in the *C. jejuni* images were specific to the presence of either silver or copper oxide NPs without compromising or disrupting the membrane integrity. In summary, these findings further demonstrate the potential usefulness of the *G. sessile*-derived metallic NPs as an alternative antimicrobial method for combating the foodborne pathogen *C. jejuni*.

## 3. Discussion

Campylobacteriosis is recognized as a significant foodborne problem with a reported incidence of approximately 550 million individuals worldwide. Data from national and international health authorities in low- and lower-middle-income countries report *Campylobacter* infections to be the main causative agent of diarrhea in children [28,29,38]. Among the species contributing to campylobacteriosis, *C. jejuni* is the most associated with symptoms of gastroenteritis in humans and is responsible for postinfectious autoimmune disorders such as Guillain–Barré and Miller Fisher syndromes. Moreover, *C. jejuni* has previously been implicated in the development of other chronic inflammatory conditions of the gastrointestinal tract, such as Crohn’s disease and ulcerative colitis [28,38].

As a complication in the treatment of disease, the prevalent antimicrobial resistance among *C. jejuni* strains has contributed to prolonged illness and increased transmission [39,40,41,42]. Recent studies have highlighted that the dramatic rise in antimicrobial resistance has been attributed to factors such as bacterial genome flexibility [43]. Furthermore, the mobility of resistance genes through horizontal gene transfer exacerbates this problem, leading to the rapid spread of multidrug-resistant strains in both clinical and agricultural settings [39,44,45]. Given the diverse disease spectra and the prevailing incidence of infections and antimicrobial resistance in *C. jejuni* strains, these observations have highlighted the imperative need for efficient and targeted infection strategies against this pathogen.

As alternative antimicrobial strategies to inhibit the growth of bacterial pathogens, nanomaterials have previously been used for combating multidrug-resistant pathogens [3,4,46]. Specifically, metallic NPs have demonstrated pathogen-killing properties, and their antimicrobial activity can be enhanced by combining them with other compounds. However, NP synthesis has a significant drawback, since the various chemicals to obtain these nanomaterials have toxicity, instability, and lower biocompatibility for biomedical applications [47]. Thus, improved biosynthesis strategies that are both effective and environmentally friendly, using green chemistry methods, have become a priority in recent years [3,48,49].

By using green chemistry for the biosynthesis of NPs, the present study employed the fungal species *G. sessile* due to its easy acquisition, management, high growth yields, and simplicity in the synthesis of metallic NPs that inhibit the enteric and opportunistic bacterial pathogens *E. coli* and *P. aeruginosa* [22,23]. Despite several studies having documented the use of metallic NPs to inhibit *C. jejuni* growth [50,51,52,53], these reports were based on conventional chemical and physical methods by employing toxic and unsafe reagents for the environment with, high demands for energy and resource consumption. To develop environmentally friendly biological methods, the present study is the first report documenting the use of green chemistry for the novel synthesis of *G. sessile*-derived metallic NPs to inhibit *C. jejuni*. In particular, the green chemistry methodology developed and optimized in this study is simple, reproductible, and cost-effective, and yielded silver and copper oxide NPs with high stability, dispersion, and suitability for biological applications.

In the synthesis of the metallic NPs in this study, meticulous control was implemented over pH and incubation temperature, which are parameters that influence the size, morphology, and surface charge of NPs [54]. To achieve the desired outcomes, the modification implemented in this study to an alkaline pH under a specific temperature during the synthesis of the metallic NPs resulted in an enhanced *in vitro* antibacterial effect against the *C. jejuni* when compared to neutral pH [22]. The permanent change in the color of the reactions indicated successful NP biosynthesis using a specific pH value with both precursor salts, and demonstrated a synthesis method that produced stable NPs comparable to previous reports [22,23]. Increasing the pH during synthesis enhanced the antimicrobial efficacy and environmental compatibility, since this type of synthesis resulted in stable particles, which can eventually be employed *in vivo* as a potential application to reduce the levels of *C. jejuni* in the poultry reservoir. Given the importance of the NPs’ suspension stability for obtaining effective antimicrobial activity, this work used the intracellular aqueous extract of *G. sessile* to replace the need for chemical stabilizers, which are commonly used in conventional reduction synthesis [9]. An additional observation was that both types of NPs exhibited a quasi-spherical shape and were embedded within an organic matrix (Figure 2B,D), which was derived from the *G. sessile* extract known to contain high levels of polysaccharides [20]. Essential metabolomic characteristics, including the high levels of polysaccharides in the organic matrix in the intracellular aqueous extract of *G. sessile* [20], contributed to the stabilizers for the optimal synthesis of small-sized silver and copper oxide NPs. The small size of these NPs is crucial for their effective antibacterial activity, as it allows for a greater surface-area-to-volume ratio, enhancing their interaction with bacterial cells. Consequently, these small-sized NPs act as potent inhibitors of bacterial growth.

Previous work has indicated that the chemical synthesis of silver NPs effectively eliminates foodborne bacterial pathogens by disrupting bacterial cell membranes and interfering with the cellular metabolism [55,56]. The *G. sessile*-derived silver NPs, synthesized using an eco-friendly method, demonstrated effective antimicrobial activity against *C. jejuni* at similar MIC values as those inhibiting the growth of *E. coli* and the opportunistic human pathogens *P. aeruginosa* and *Staphylococcus aureus* [23]. The first approach used to observe how both types of NPs act (Figure 3A) showed results that, at first, indicated that there was antibacterial activity, but also indicated that it was necessary to control better factors such as resuspension of NPs in a liquid medium, as well as reducing the initial bacterial inoculum for subsequent tests in liquid. To achieve this safely, an essential step performed in this study was to remove the aqueous phase in the final phase of the synthesis to ensure that the NPs could be resuspended in a medium suitable for bacterial culturing without depleting any nutrients during subsequent assay incubations. Copper and copper oxide NPs have also been used previously to inhibit various pathogens with ideal MICs based on various mechanisms of inhibition [8]. The green synthesis of copper oxide NPs resulted in lower MIC values when compared with previous reports, documenting the use of *G. sessile* particles against *E. coli* and *P. aeruginosa* [22]. The antibacterial effect against *C. jejuni* growth in this research after treatment with biologically synthetized NPs at low concentrations was consistent with values using chemically synthesized NPs, as has been reported in previous studies [50,51,52,53].

To understand the mechanisms of nanoparticle action, the interactions between silver and copper oxide NPs and *C. jejuni* were further examined using an ROS test and transmission electron microscopy. After examining the *C. jejuni* culture without NPs added and representing the continuously generated species as part of the metabolism [57], the levels of oxidant formation were comparable to those obtained after incubation with 0.5 µg/mL of silver NPs (Figure 4A). Given the short lifespan of reactive species [40], lower levels of ROS would be expected at the highest concentrations of silver NPs due to a potential antioxidant effect and, consequently, ROS quenching, as has been documented previously for silver NPs derived from herbal and fungal crude extracts [58]. These observations agreed with previous reports, indicating a prevalent association of silver NPs with the bacterial outer cell membrane in other Gram-negative bacterial pathogens, including *Escherichia coli* and *Pseudomonas aeruginosa* [59,60]. In the case of the interaction of the bacteria with copper oxide NPs, no significant differences were observed between the treatments with different concentrations of NPs or with the control that did not contain NPs. This can be explained because, as mentioned, they were still alive after 6 h of interaction of the bacteria with the copper oxide NPs. This is a phenomenon that may make sense in support of a slower bactericidal effect caused by the copper NPs with the detection of prolonged levels of ROS generation [61,62,63].

The present study documented the visualization of the interaction of both types of green-synthesized metallic NPs with the outer cell membrane in *C. jejuni* for the first time. Additionally, the representative images also demonstrated the apparent intracellular internalization of the copper oxide NPs. The distinct interaction of the silver and copper oxide NPs with *C. jejuni* highlights potential distinct mechanisms of action for both types of metallic NPs. In detail, silver NPs exhibited rapid bactericidal effects, coinciding with minimal ROS production, and indicated a rapid bactericidal action within 3 h of incubation. The mode of action of the silver NPs was likely due to their ability to quickly disrupt the bacterial membrane and internalize, causing immediate cellular stress and damage [58]. By contrast, copper oxide NPs showed a slower bactericidal effect and resulted in the interaction of the copper oxide NPs with the bacterial membrane and subsequent internalization with sustained oxidative stress over time. These findings agree with previous studies in which bactericidal effects were reported after longer incubation times with copper oxide NPs against pathogens such as *E. coli*, *Klebsiella oxytoca*, and *S. aureus* [61,62,63]. These observed differences underscore the unique properties of each type of NP, contributing to their respective antibacterial efficacies and mechanisms.

In summary, the green synthesis of NPs holds significant potential as an alternative method to inhibit the growth of the foodborne bacterial pathogen *C. jejuni*. Utilizing biological organisms or natural extracts for NP synthesis reduces the dependence on hazardous chemicals and enhances the biocompatibility and stability of the NPs for biological applications. This eco-friendly approach contributes to sustainable and innovative antimicrobial strategies, addressing the growing concern of antimicrobial resistance and the need for safe food preservation methods. By integrating green chemistry principles, this method supports the development of effective antimicrobial agents that are environmentally benign and sustainable.

## 4. Materials and Methods

### 4.1. Growth Conditions and Extract Preparation of the Fungus G. sessile

The fungus *G. sessile* (strain CDM1-Gs1) was obtained from the laboratory strain collection in the Microbiology Department (CICESE, Ensenada, Mexico). The fungus was grown in Petri dishes with potato dextrose agar medium and incubated at 30 °C for 4 days, as in previous studies [22,23]. Subsequently, seven plugs of the fungal mycelium were taken from the plates and placed into a 250 mL Erlenmeyer flask with 100 mL of potato dextrose broth (Merck Millipore, Mexico City, Mexico), and were further incubated at 30 °C with 120 rpm shaking. After incubation for seven days, an intracellular extract of *G. sessile* was prepared by filtering the fungal mycelium using a vacuum pump and a 0.45 µm pore size Whatman nylon filter (Sigma-Aldrich, St. Louis, MI, USA), then subsequently washing the collected mycelium with deionized water [22]. The aqueous fungal extract was then prepared by maceration with an agate mortar and pestle in deionized water using a 1:1 proportion (*w*/*v*), followed by filtration with an 0.2 µm Whatman nylon filter (Sigma-Aldrich) and subsequent storage at 4 °C until further use.

### 4.2. Biosynthesis and Characterization of the Metallic NPs

A stock suspension of the metal precursor salts, 10 mM copper sulfate pentahydrate (CuSO_4_∙5H_2_O) or 1 mM silver nitrate (AgNO_3_), was used to synthesize the biogenic NPs. For the synthesis of copper oxide NPs and silver NPs, a ratio of 1:3 (*v/v*) of the aqueous fungal extract with either CuSO_4_∙5H_2_O or AgNO_3_ was used. The pH of the suspension was adjusted to pH 8 using sodium hydroxide at 100 mM and was further incubated at 60 °C for three days. A subsequent characterization of the synthetized NPs was performed by measuring the absorbance in the 200 to 700 nm range using a PerkinElmer UV-Vis spectrophotometer (Waltham, MA, USA). The absorbance peak for copper oxide NPs was expected to be between 220 and 250 nm, while for silver NPs, between 400 and 480 nm [64]. The hydrodynamic diameter, size, and zeta potential of the synthesized NPs were determined using a Zetasizer Nano ZS instrument (Malvern Panalytical Inc., Westborough, MA, USA).

### 4.3. Growth and Propagation of the Bacterial Foodborne Pathogen C. jejuni

The *C. jejuni* strain ATCC 33560 [65] was purchased from the American Type Culture Collection (Manassas, VA, USA) and cultured on Oxoid Anaerobic Basal Agar CM0972 (ABA) (Thermo Fisher Scientific, Pittsburgh, PA, USA) supplemented with 5% sterile defibrinated sheep blood (DIBICO, Mexico City, Mexico). The *C. jejuni* cultures were routinely propagated by incubation in sealed plastic bags at 42 °C for 48 h under microaerophilic conditions, which consisted of 10% hydrogen and 10% carbon dioxide balanced with nitrogen (INFRA, Ensenada, Baja California, Mexico).

### 4.4. In Vitro Assay to Assess Growth Inhibition of C. jejuni

To evaluate the inhibitory effect of the NPs against *C. jejuni*, a culture of *C. jejuni* was initially grown on ABA for 2 days at 42 °C under microaerophilic conditions and was then used to prepare a bacterial cell suspension in Oxoid Bolton Broth CM0983 (Thermo Fisher Scientific). As a first approach to test the antibacterial activity of silver and copper oxide NPs, a culture was carried out in a 96-well plate. An initial culture with an optical density of 0.5 at 600 nm was used in this test. Nanoparticles were in the aqueous phase, as they were initially obtained, and the test was conducted using a mixture comprising 50 μL of Bolton Broth culture medium, 50 μL of NPs at different concentrations, and 100 μL of the bacterial suspension. This setup was employed to evaluate the immediate inhibitory effects of the NPs on *C. jejuni*, allowing for a rapid assessment of antibacterial activity under controlled conditions. Subsequently, the NPs were concentrated by centrifugation at 252 ×g for 10 min to remove the aqueous medium in which they were suspended, and they were further resuspended in Bolton Broth at a concentration of 26 μg/mL for the silver NPs and 1 mg/mL for the copper oxide NPs. Growth inhibition assays were performed using T-25 vented cell culture flasks (Thermo Fisher Scientific) after the addition of the *C. jejuni* cell suspension, which was adjusted to an optical density of 0.4 at 600 nm, and various concentrations of either the silver NPs, ranging from 0.08 μg/mL to 13 μg/mL, or copper oxide NPs, ranging from 1 μg/mL to 20 μg/mL. Separate T-25 flasks containing *C. jejuni* without NPs served as the negative control. The individual T-25 flasks were sealed in plastic bags and further incubated for 48 h at 42 °C without shaking under a microaerophilic atmosphere. After the incubation period, the growth of *C. jejuni* was determined after plating serial dilutions of the cell suspension on Oxoid Campylobacter Blood-Free Selective Agar Base CM0739B (Thermo Fisher Scientific), followed by subsequent quantification of colony-forming units after incubation for 48 h at 42 °C. The minimum inhibitory concentration was defined as the concentration of NPs where there was no bacterial growth, as determined in previous studies [22].

### 4.5. In Vitro Test to Determine ROS Generation by C. jejuni

To assess the generation of ROS by *C. jejuni*, a modified version of a previously described protocol was used [22]. In a 96-well plate, a suspension of *C. jejuni* was seeded at a density of 1 × 10^5^ cells per well and exposed to different concentrations of silver NPs (10 μg/mL, 6 μg/mL, 3 μg/mL, 1 μg/mL, and 0.5 μg/mL), and copper oxide NPs (100 μg/mL, 50 μg/mL, 20 μg/mL, 10 μg/mL, and 5 μg/mL), followed by incubation at 42 °C for 3 h. As a control for ROS generation, bacterial cultures were incubated with 1 mM H_2_O_2_, while negative controls comprised cells without NPs. Following NP treatment, the *C. jejuni* cells underwent triple washing with 200 μL of 1× phosphate-buffered saline (PBS). Subsequently, the 1× PBS was removed, and the cells were incubated at 42 °C in darkness with 100 μL of 45 μM of 2′,7′-dichlorofluorescein diacetate (D6883, Merck Millipore) for 60 min [35]. The fluorescence (λ_ex_ = 485 nm and λ_em_ = 520 nm) was measured using a Cary Eclipse Fluorescence Spectrophotometer (Agilent Technologies, Santa Clara, CA, USA).

### 4.6. Transmission Electron Microscopy

Transmission electron microscopy was performed to characterize the synthetized NPs. Briefly, 5 µL of each nanoparticle was deposited onto formvar/carbon-coated copper grids and allowed to air-dry. Samples were analyzed using a Hitachi H7500 transmission electron microscope (Hitachi Ltd., Tokyo, Japan) at 100 kV. To generate the size graphs of the NPs, images of the NPs were captured with ImageJ software version 1.8.0 [66], and the particle size data were then analyzed using R software, version 4.1.3 [67]. To subsequently assess the effect on *C. jejuni* after nanoparticle exposure, *C. jejuni* cultures were incubated in the presence of the copper oxide NPs and silver NPs at the minimum inhibitory concentrations of 10 μg/mL and 6.7 μg/mL, respectively, and were then prepared for microscopy analysis as previously described [37]. After incubation for 48 h at 42 °C under microaerophilic conditions, the samples were centrifuged for 5 min at 252 ×g for the fixation and dehydration processes, followed by 10 min at 1006× *g* during infiltration. After concentration, the pellets were fixed with 2% glutaraldehyde in 0.05 M phosphate buffer for 30 min at room temperature. The glutaraldehyde-fixed *C. jejuni* were then washed with 1× PBS, post-fixed with 1% osmium tetroxide for 2 h at 4 °C, and then dehydrated with an ethanol series for 15 min at each concentration (15, 30, 50, and 75%) and for 30 min with 100% ethanol. Subsequently, the samples were infiltrated in a resin–ethanol series at various concentrations (15, 30, 50, 75, and 100%) for 15 min at each resin concentration and left overnight with 100% Spurr resin (Science Services, Copenhagen, Denmark). Finally, the samples were placed on molds containing 100% Spurr resin and polymerized at 60 °C for 24 h. After cooling, the samples were sectioned using a Leica Ultracut R ultramicrotome (Leica Microsystems Inc., Buffalo Grove, IL, USA). Thin dissections of 70 nm were mounted on 75-mesh copper grids coated with formvar/carbon and analyzed under a transmission electron microscope (Hitachi H7500) operated at 100 kV. Sections were examined without post-staining for better detection of NPs.

## 5. Conclusions

This is the first report in which green synthesized NPs have been successfully tested as antibacterial agents against the foodborne pathogen *C. jejuni*. The faster inhibitory action of silver NPs is believed to result from their interaction with the bacterial membrane, leading to rapid disruption and cell death. In contrast, copper oxide NPs exhibited a slower inhibitory effect, potentially due to the incorporation of copper into bacterial metabolic pathways, thereby exerting slower, but effective, antimicrobial activity. Notably, the minimum inhibitory concentrations of these NPs are low, underscoring their efficacy.

In summary, this work highlights the potential of green-synthesized metallic NPs derived from fungi as powerful antimicrobial agents against *C. jejuni*. This innovative approach represents a promising strategy for developing effective antimicrobial treatments against this challenging pathogen.

## Data Availability

The raw data supporting the conclusions of this article will be made available by the authors upon request.
